# Association of Remnant Cholesterol with Platelet Reactivity in Coronary Artery Disease Patients Receiving PCI

**DOI:** 10.5334/gh.1475

**Published:** 2025-10-15

**Authors:** Menglu Liu, Jiawen Li, Kailun Yan, Kexin Zhang, Pei Zhu, Xiaofang Tang, Deshan Yuan, Yuejin Yang, Runlin Gao, Jinqing Yuan, Xueyan Zhao

**Affiliations:** 1 National Clinical Research Center for Cardiovascular Diseases, State Key Laboratory of Cardiovascular Disease, Fu Wai Hospital, National Center for Cardiovascular Diseases, Chinese Academy of Medical Sciences and Peking Union Medical College, Beijing, China

**Keywords:** Cholesterol, platelet function tests, percutaneous coronary intervention, bleeding, ischemia

## Abstract

**Background:**

Remnant cholesterol (RC) has received increasing attention and shown to be associated with bleeding and ischemic events in clinical research; however, the mechanisms remain incompletely understood.

**Aim:**

To investigate the relationship between RC and platelet reactivity in patients with coronary artery disease (CAD) undergoing percutaneous coronary intervention (PCI) who received dual antiplatelet therapy with aspirin and clopidogrel.

**Methods:**

A total of 10,724 consecutive PCI patients in China from January 2013 to December 2013 were enrolled. 6,633 patients had the results of thromborlastogram for analysis. Low on-treatment platelet reactivity (LTPR) and high on-treatment platelet reactivity (HTPR) were defined as adenosine diphosphate-induced platelet maximum amplitude of thromborlastogram <31 mm and >47 mm, respectively.

**Results:**

A total of 6,633 PCI patients (mean age, 58.20 ± 10.2 years; male, 77.5%) were finally enrolled. When RC was used as a continuous variable, the multivariate logistic regression showed that RC concentration was negatively associated with LTPR (OR: 0.761, 95% CI 0.609–0.950) and positively associated with HTPR (OR: 1.461, 95% CI 1.151–1.855). For RC quartiles, compared to the lowest quartile (Q1), quartiles 3 and 4 were negatively associated with LTPR (OR_Q3_: 0.853, 95% CI 0.735–0.990; OR_Q4_: 0.840, 95% CI 0.707–0.999). Meanwhile, higher quartiles of RC (Q2, Q3, Q4) were positively associated with HTPR (OR_Q2_: 1.193, 95% CI 1.015–1.402; OR_Q3_: 1.356, 95% CI 1.152–1.596; OR_Q4_: 1.404, 95% CI 1.164–1.694).

**Conclusions:**

We reported that RC was associated with clopidogrel-related platelet reactivity in patients undergoing PCI received dual antiplatelet therapy. These results suggest an interaction between lipid and thrombosis, and remind us pay attention to RC levels in PCI patients.

**Key findings:**

In the large-scale (n = 6,633) and real-world study, we revealed that RC may modify the platelet reactivity to influence the risk of bleeding and ischemia in PCI patients. Our study firstly reported the relationship between RC and clopidogrel-related platelet reactivity in patients undergoing PCI received dual antiplatelet therapy. The findings may verify the complex interaction between lipid and thrombosis and suggest that RC may be a potential marker associated with platelet reactivity, which warrants further investigation in future studies.

**Figure:**
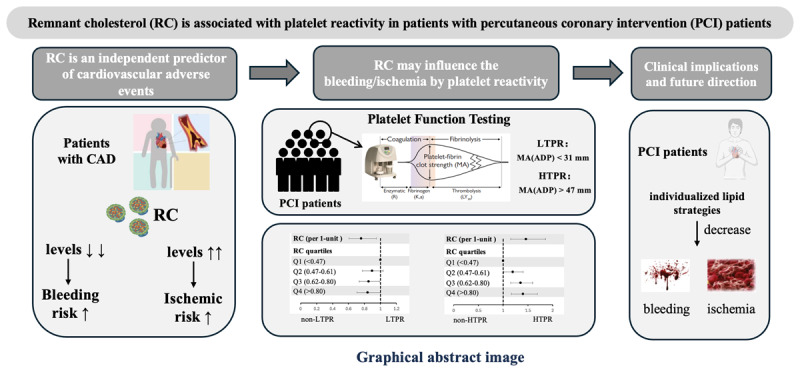


## 1. Introduction

In recent years, the role of remnant cholesterol (RC) in cardiovascular disease has gained increasing attention. Studies have demonstrated that elevated RC is an independent risk factor for coronary artery disease (CAD) and is associated with a higher risk of adverse cardiovascular events (1,2,3). Reducing RC may provide protective benefits in lowering CAD risk; Interestingly, including findings from our previous research, extremely low levels of RC were also associated with bleeding events (4,5,6). However, the underlying mechanisms of this association remain unclear.

Patients with CAD commonly take antiplatelet agents to reduce the risk of thrombotic events, but antiplatelet therapy also probably increases the risk of bleeding. Clinicians face the challenge of balancing ischemic and bleeding risks in patients following percutaneous coronary intervention (PCI) (7). Measuring platelet reactivity can reflect the effectiveness of antiplatelet medication, with low on-treatment platelet reactivity (LTPR) being associated with bleeding risk and high on-treatment platelet reactivity (HTPR) being linked to ischemic risk (8,9). Platelet reactivity is influenced by multiple factors, including genetics, drug effects, and clinical characteristics (10). Some studies suggest that lipids and thrombosis form a complex interactive network that mutually influences one another (11). Therefore, we hypothesize that different concentrations of RC may alter ischemic and bleeding risks by affecting platelet reactivity. To date, no studies have investigated the relationship between RC and platelet reactivity.

In this study, we aim to explore the effect of RC on clopidogrel-related platelet reactivity in patients undergoing PCI received dual antiplatelet therapy, examining whether RC influences the platelet reactivity. We seek to elucidate the potential mechanisms underlying bleeding and ischemic events in this population, providing new insights and directions for clinicians to personalize lipid-lowering and antiplatelet therapy in clinical practice.

## 2. Methods

### 2.1. Study design and population

**Figure 1:**
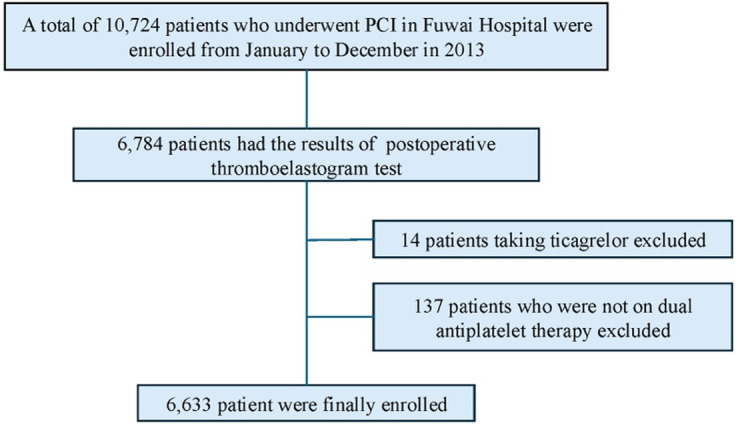
Flowchart of the study. PCI, percutaneous coronary intervention.

This study was a prospective, observational, single-center investigation conducted in a real-world setting. Between January and December 2013, 10,724 PCI patients were consecutively recruited at Fu Wai Hospital, Beijing, China. A total of 6,633 patients who had thromboelastography testing and received clopidogrel were included in the final analysis (Figure 1).

In this study, all patients underwent PCI in accordance with the current practice guidelines in China. For those undergoing selective PCI without prior long-term aspirin and clopidogrel therapy, a loading dose of 300 mg of aspirin and 300 mg of clopidogrel was administered orally at least 24 hours prior to the procedure. Postoperatively, all patients received daily aspirin (100 mg) in combination with clopidogrel (75 mg) for at least 12 months.

This study was conducted in accordance with the principles of the Helsinki Declaration and received approval from the ethics committee of Fu Wai Hospital, Beijing, China (Approval number: 2013–449). Written informed consent was obtained from all participants.

### 2.2. Platelet Reactivity Measurement and Definition

Platelet reactivity was assessed using TEG platelet mapping, with adenosine diphosphate (ADP)-induced maximum amplitude [MA(ADP)] reflecting fibrin clot strength under ADP stimulation. All patients underwent TEG testing in the morning after PCI, within 24 hours post-procedure. Fasting venous blood samples (2 ml) were drawn from patients in a supine position. The TEG 5000 Thrombelastograph Hemostasis Analyzer system (Haemonetics Corporation, MA, USA) was employed to evaluate platelet reactivity. Whole blood samples were anticoagulated with 3.2% sodium citrate, and all procedures, from blood collection to analysis, were completed within two hours. HTPR and LTPR were defined according to established cut-off values: MA(ADP) > 47 mm for HTPR and MA(ADP) < 31 mm for LTPR (12,13).

### 2.3. Laboratory Measurement

All patients underwent venous blood sampling upon admission after fasting for more than 12 hours. Triglycerides (TG), total cholesterol (TC), low-density lipoprotein cholesterol (LDL-C), and high-density lipoprotein cholesterol (HDL-C) levels were measured using an automatic biochemistry analyzer (Hitachi 7150, Tokyo, Japan), under strict quality control protocols in the Clinical Chemistry Department of Fu Wai Hospital. Specifically, plasma TC was measured by the cholesterol oxidase-phenol aminophenazone method, plasma TG by the glycerol-3-phosphate oxidase-phenol aminophenazone method, plasma HDL-C by a chemically modified enzyme method, and plasma LDL-C by a selective precipitation method. RC was calculated as RC = TC – HDL-C – LDL-C (2,14).

### 2.4. Statistical Analysis

Continuous data are presented as mean ± standard deviation or median (interquartile range). Categorical variables are expressed as percentages. Group comparisons for continuous variables were performed using one-way analysis of variance, while categorical variables were compared using the chi-squared test.

RC concentrations were modeled as both continuous variables and quartiles. According to the quartiles of RC, patients were stratified into four groups: the lowest quartile 1 (Q1: RC ≤ 0.46, n = 1,677), the lower quartile 2 (Q2: 0.46 < RC ≤ 0.61, n = 1,620), the higher quartile 3 (Q3: 0.61 < RC ≤ 0.80, n = 1,659) and the highest quartile 4 (Q4: RC > 0.80, n = 1,677). Potential factors linked to HTPR and LTPR were initially identified using univariable logistic regression and included in multivariable analysis. The association between RC and platelet reactivity was assessed using multivariable logistic regression, with Odds Ratios (OR) and 95% Confidence Intervals (CI) as effect estimates. Using multivariate restricted cubic spline to evaluate non-linear association of RC with LTPR and HTPR. Subgroup analyses were performed for robustness, and interaction P-values were calculated. Subgroups were stratified based on age (≥ 65 years), acute coronary syndrome (ACS) at admission, hypertension, and statin use.

A two-sided P-value of <0.05 was considered statistically significant. All analyses were performed using SPSS software version 26.0 (IBM Corp., Armonk, New York, USA) and R Programming Language version 4.0.3 (R Foundation for Statistical Computing, Vienna, Austria).

## 3. Results

### 3.1. Baseline Characteristics

Finally, there were 6,633 eligible patients included (Figure 1) for analysis. The mean age was 58.20 ± 10.25 years and 5,142 (77.5%) were men. Table 1 presents the characteristics of participants stratified by RC quartiles: Q1 (RC ≤ 0.46, n = 1,677), Q2 (0.46 < RC ≤ 0.61, n = 1,620), Q3 (0.61 < RC ≤ 0.80, n = 1,659), and Q4 (RC > 0.80, n = 1,677). Among the four groups, individuals in Q1 had significantly the highest proportion of male, the oldest age, the lowest BMI, the lowest prevalence of acute coronary syndrome, smoking history, hyperlipidemia, HTPR, the use of β- blockers, the highest prevalence of prior myocardial infarction, prior PCI, LTPR, the highest baseline levels of LVEF, HDL-C as well as the lowest levels of hemoglobin, platelet, LDL-C, TC, TG, glucose and MA(ADP) (Table 1). In addition, Table S1 depicts the comparisons of baseline characteristics based on different classifications of platelet reactivity.

**Table 1:** Baseline characteristics of patients according to the quartiles of remnant cholesterol.


PARAMETER	Q1 N = 1677	Q2 N = 1620	Q3 N = 1659	Q4 N = 1677	*P*-VALUE

**DEMOGRAPHICS**					

Sex [male, n(%)]	1342 (80.02)	1256 (77.53)	1280 (77.15)	1264 (75.37)	0.014

Age, year	60.120 ± 10.068	59.010 ± 10.222	57.760 ± 10.171	55.920 ± 10.072	<0.001

BMI, kg/m^2^	25.402 ± 3.181	25.857 ± 3.223	26.068 ± 3.135	26.601 ± 3.019	<0.001

**CLINICAL CHARACTERISTICS**					

ACS, *n* (%)	899 (53.61)	907 (55.99)	964 (58.11)	1025 (61.12)	<0.001

Smoking history, *n* (%)	902 (53.79)	918 (56.67)	960 (57.87)	989 (58.97)	0.017

Hyperlipidemia, *n* (%)	1036 (61.78)	1087 (67.10)	1170 (70.52)	1235 (73.64)	<0.001

Hypertension, *n* (%)	1058 (63.09)	1076 (66.42)	1064 (64.14)	1117 (66.61)	0.087

Diabetes mellitus, n (%)	498 (29.70)	516 (31.85)	475 (28.63)	537 (32.02)	0.091

COPD, *n* (%)	35 (2.09)	32 (1.98)	44 (2.65)	36 (2.15)	0.561

Family history of CHD, *n* (%)	382 (22.78)	391 (24.14)	430 (25.92)	437 (26.06)	0.086

Cerebrovascular disease history, *n* (%)	179 (10.67)	174 (10.74)	189 (11.39)	145 (8.65)	0.054

Peripheral vascular disease, *n* (%)	57 (3.40)	34 (2.10)	50 (3.01)	49 (2.92)	0.152

Prior myocardial infarction, *n* (%)	351 (20.93)	333 (20.56)	343 (20.68)	286 (17.05)	0.013

Prior PCI, *n* (%)	453 (27.01)	428 (26.42)	377 (22.72)	370 (22.06)	<0.001

Prior CABG, *n* (%)	74 (4.41)	61 (3.77)	54 (3.25)	82 (4.89)	0.087

**LABORATORY VARIABLES**					

LVEF, %	63.627 ± 6.909	62.776 ± 6.972	62.608 ± 7.602	62.671 ± 7.189	<0.001

Hemoglobin, g/L	141.187 ± 15.445	142.423 ± 14.740	142.69 ± 14.931	144.609 ± 15.382	<0.001

PLT, 10^9^/L	194.874 ± 50.084	202.890 ± 52.960	208.90 ± 56.496	214.355 ± 57.358	<0.001

MPV, fL	10.579 ± 0.887	10.620 ± 0.922	10.619 ± 0.924	10.592 ± 0.921	0.497

LDL-C, mmol/L	2.248 ± 0.785	2.370 ± 0.826	2.547 ± 0.864	2.771 ± 0.992	<0.001

HDL-C, mmol/L	1.157 ± 0.296	1.045 ± 0.266	0.988 ± 0.237	0.921 ± 0.218	<0.001

TC, mmol/L	3.743 ± 0.890	3.956 ± 0.935	4.237 ± 0.956	4.822 ± 1.130	<0.001

Triglyceride, mmol/L	1.070 ± 0.327	1.384 ± 0.345	1.729 ± 0.406	2.914 ± 1.426	<0.001

Glucose, mmol/L	5.913 ± 1.860	6.043 ± 1.849	6.049 ± 1.873	6.365 ± 2.158	<0.001

eGFR, ml/min	91.362 ± 13.889	91.566 ± 14.460	91.683 ± 15.010	92.061 ± 15.492	0.573

**MEDICATION**					

statin	1614 (96.24)	1553 (95.86)	1609 (96.99)	1610 (96.00)	0.328

β-blockers	1481 (88.31)	1473 (90.93)	1517 (91.44)	1539 (91.77)	0.002

CCB	844 (50.33)	816 (50.37)	803 (48.40)	874 (52.12)	0.203

**PLATELET REACTIVITY**					

MA(ADP), mm	34.257 ± 16.963	35.729 ± 17.674	36.761 ± 17.924	36.988 ± 17.773	<0.001

LTPR	677 (40.37)	612 (37.78)	600 (36.17)	605 (36.08)	0.035

HTPR	1237 (73.76)	1137 (70.19)	1108 (66.79)	1110 (66.19)	<0.001


BMI, body mass index; ACS, acute coronary syndrome; COPD, chronic obstructive pulmonary disease; CHD, coronary heart disease; PCI, percutaneous coronary intervention; CABG, coronary artery bypass graft; LVEF, left ventricle ejection fraction; PLT, platelet count; MPV, mean platelet volume; LDL-C, low-density lipoprotein cholesterol; HDL-C, high-density lipoprotein cholesterol; TC, total cholesterol; eGFR, estimated glomerular filtration rate; CCB, calcium channel blocker; MA(ADP), adenosine diphosphate (ADP)-induced platelet maximum amplitude; LTPR, low on-treatment platelet reactivity; HTPR, high on-treatment platelet reactivity.

### 3.2. Association between remnant cholesterol and platelet reactivity

In the total population, there were 2,494 (37.60%) presented as LTPR, 2,098 (31.63%) as NTPR and 2,041 (30.77%) as HTPR.

**Figure 2:**
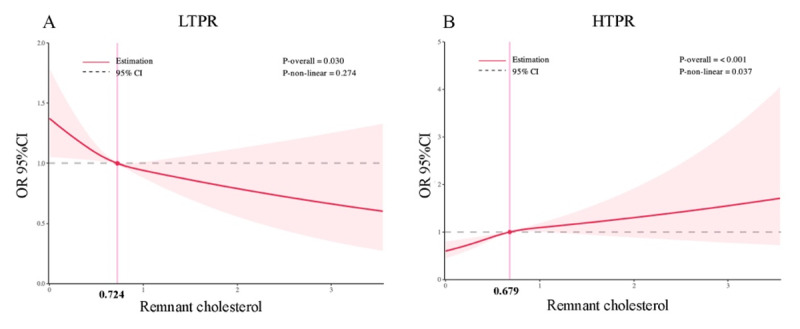
Restricted cubic spline curves of remnant cholesterol for LTPR and HTPR. CI, confidence interval; OR, odd ratio.

For LTPR, when RC was a continuous variable, adjusting for significant covariates in the univariate model, the multivariable logistic regression analysis showed that RC concentration was independently and negatively with LTPR (OR: 0.761, 95% CI 0.609–0.950; p = 0.016) (Table 2). The RCS regression model also showed a linearly decreasing relationship between RC concentrations and LTPR (P for all = 0.030; p for nonlinear = 0.274) (Figure 2A).

**Table 2:** Logistic regression for LTPR.


VARIABLES	UNIVARIATE REGRESSION		MULTIVARIATE REGRESSION		MULTIVARIATE REGRESSION	
		
	OR (95% CI)	*P*-VALUE	ADJUSTED OR (95% CI)	*P*-VALUE	ADJUSTED OR (95% CI)	*P*-VALUE

Sex	0.509 (0.448–0.579)	<0.001	1.114 (0.938–1.323)	0.219	1.082 (0.912–1.283)	0.366

Age	0.983 (0.978–0.988)	<0.001	0.993 (0.986–1.000)	0.038	0.994 (0.987–1.000)	0.058

BMI	0.999 (0.984–1.015)	0.915	—	—	—	—

ACS	0.903 (0.817–0.998)	0.046	1.058 (0.870–1.286)	0.574	0.963 (0.866–1.071)	0.488

Smoking history	1.336 (1.208–1.478)	<0.001	1.136 (0.952–1.355)	0.157	1.012 (0.898–1.139)	0.849

Hyperlipidemia	1.016 (0.913–1.131)	0.765	—	—	—	—

Hypertension	0.910 (0.820–1.010)	0.076	—	—	—	—

COPD	0.934 (0.665–1.313)	0.696	—	—	—	—

Family history of CHD	1.019 (0.908–1.143)	0.753	—	—	—	—

Cerebrovascular disease	0.830 (0.703–0.981)	0.029	0.917 (0.771–1.091)	0.329	0.915 (0.769–1.088)	0.315

Peripheral vascular disease	0.990 (0.735–1.334)	0.947	—	—	—	—

Prior myocardial infarction	1.138 (1.006–1.288)	0.040	1.041 (0.902–1.202)	0.579	1.041 (0.902–1.202)	0.581

Prior PCI	1.179 (1.051–1.322)	0.005	1.139 (1.005–1.290)	0.042	1.139 (1.005–1.291)	0.041

Prior CABG	0.953 (0.741–1.227)	0.711	—	—	—	—

LVEF	1.009 (1.002–1.016)	0.011	1.009 (1.001–1.017)	0.027	1.009 (1.001–1.017)	0.027

Hemoglobin	1.029 (1.026–1.033)	<0.001	1.028 (1.024–1.033)	<0.001	1.029 (1.024–1.033)	<0.001

PLT	0.998 (0.997–0.999)	<0.001	0.999 (0.998–1.000)	0.008	0.999 (0.998–1.000)	0.011

MPV	1.028 (0.973–1.085)	0.326	—	—	—	—

LDL-C	0.875 (0.827–0.927)	<0.001	0.741 (0.586–0.938)	0.013	0.861 (0.720–1.029)	0.100

HDL-C	1.003 (0.835–1.205)	0.975	—	—	—	—

TC	0.895 (0.853–0.938)	<0.001	1.150 (0.926–1.428)	0.207	1.002 (0.853–1.176)	0.983

Triglyceride	1.000 (0.954–1.048)	0.994	—	—	—	—

Glucose	0.956 (0.931–0.981)	<0.001	0.951 (0.925–0.977)	<0.001	0.949 (0.923–0.976)	<0.001

eGFR	1.007 (1.004–1.011)	<0.001	0.999 (0.994–1.003)	0.599	0.999 (0.994–1.003)	0.609

statin	0.837 (0.646–1.083)	0.176	—	—	—	—

β-blockers	0.916 (0.773–1.084)	0.307	—	—	—	—

CCB	1.101 (0.996–1.216)	0.059	—	—	—	—

RC, mmol/L	0.865 (0.755–0.991)	0.037	0.761 (0.609–0.950)	0.016	—	—

RC	0.035					

Q1	**Reference**		—	—	**Reference**	

Q2	0.897 (0.780–1.032)	0.127	—	—	0.896 (0.775–1.036)	0.140

Q3	0.837 (0.728–0.962)	0.013	—	—	0.853 (0.735–0.990)	0.036

Q4	0.834 (0.725–0.958)	0.011	—	—	0.840 (0.707–0.999)	0.049


BMI, body mass index; ACS, acute coronary syndrome; COPD, chronic obstructive pulmonary disease; CHD, coronary heart disease; PCI, percutaneous coronary intervention; CABG, coronary artery bypass graft; LVEF, left ventricle ejection fraction; PLT, platelet count; MPV, mean platelet volume; LDL-C, low-density lipoprotein cholesterol; HDL-C, high-density lipoprotein cholesterol; TC, total cholesterol; RC, remnant cholesterol; eGFR, estimated glomerular filtration rate; CCB, calcium channel blocker; MA(ADP), adenosine diphosphate (ADP)-induced platelet maximum amplitude; LTPR, low on-treatment platelet reactivity; HTPR, high on-treatment platelet reactivity.

In the further analysis, based on quartiles of RC, compared to Q1, after adjusting for the same significant covariates, the multivariable logistic regression analysis showed that Q4 had the lowest risk of LTPR (OR: 0.840, 95% CI 0.707–0.999; p = 0.049), followed by Q3 (OR: 0.853, 95% CI 0.735–0.990; p = 0.036) (Table 2).

For HTPR, when RC was a continuous variable, adjusting for significant covariates in the univariate model, the multivariable logistic regression analysis showed that RC concentration was independently and positively with HTPR (OR: 1.461, 95% CI 1.151–1.855; p = 0.002) (Table 3). The RCS regression model also showed a linearly increasing relationship between RC concentrations and HTPR (P for all = 0.004; p for nonlinear = 0.146) (Figure 2B).

**Table 3:** Logistic regression for HTPR.


VARIABLES	UNIVARIATE REGRESSION		MULTIVARIATE REGRESSION		MULTIVARIATE REGRESSION	
		
	OR (95% CI)	*P*-VALUE	ADJUSTED OR (95% CI)	*P*-VALUE	ADJUSTED OR (95% CI)	*P*-VALUE

Sex	0.325 (0.288–0.366)	<0.001	0.649 (0.548–0.769)	<0.001	0.673 (0.569–0.796)	<0.001

Age	1.204 (1.019–1.029)	<0.001	1.008 (1.001–1.016)	0.024	1.008 (1.001–1.015)	0.028

BMI	0.997 (0.981–1.014)	0.750	—	—	—	—

ACS	1.178 (1.059–1.309)	0.002	1.070 (0.954–1.200)	0.250	1.069 (0.953–1.199)	0.256

Smoking history	0.595 (0.536–0.661)	<0.001	0.996 (0.872–1.136)	0.948	0.995 (0.872–1.135)	0.940

Hyperlipidemia	0.967 (0.865–1.081)	0.557	—	—	—	—

Hypertension	1.149 (1.029–1.283)	0.014	0.977 (0.868–1.100)	0.703	0.978 (0.868–1.101)	0.709

COPD	1.093 (0.771–1.549)	0.617	—	—	—	—

Family history of CHD	0.964 (0.854–1.088)	0.552	—	—	—	—

Cerebrovascular disease	1.141 (0.964–1.349)	0.124	—	—	—	—

Peripheral vascular disease	1.263 (0.934–1.707)	0.129	—	—	—	—

Prior myocardial infarction	0.776 (0.678–0.889)	<0.001	0.971 (0.834–1.132)	0.708	0.970 (0.832–1.130)	0.692

Prior PCI	0.823 (0.727–0.931)	0.002	0.908 (0.791–1.043)	0.174	0.911(0.793–1.046)	0.187

Prior CABG	0.823 (0.626–1.082)	0.163	—	—	—	—

LVEF	0.995 (0.988–1.002)	0.194	—	—	—	—

Hemoglobin	0.961 (0.958–0.965)	<0.001	0.967 (0.963–0.971)	<0.001	0.966 (0.962–0.971)	<0.001

PLT	1.005 (1.004–1.006)	<0.001	1.004 (1.003–1.005)	<0.001	1.004 (1.003–1.005)	<0.001

MPV	0.970 (0.916–1.027)	0.298	—	—	—	—

LDL-C	1.198 (1.131–1.269)	<0.001	1.434 (1.113–1.848)	0.005	1.213 (0.997–1.476)	0.054

HDL-C	1.159 (0.957–1.403)	0.132	—	—	—	—

TC	1.180 (1.125–1.239)	<0.001	0.834 (0.660–1.054)	0.129	0.968 (0.811–1.155)	0.718

Triglyceride	1.030 (0.981–1.081)	0.239	—	—	—	—

Glucose	1.063 (1.036–1.091)	<0.001	1.067 (1.038–1.098)	<0.001	1.069 (1.039–1.099)	<0.001

eGFR	0.988 (0.985–0.992)	<0.001	0.999 (0.994–1.003)	0.570	0.999 (0.994–1.003)	0.619

statin	1.370 (1.021–1.839)	0.036	1.433 (1.049–1.958)	0.024	1.433 (1.049–1.957)	0.024

β-blockers	1.113 (0.928–1.336)	0.247	—	—	—	—

CCB	0.970 (0.874–1.076)	0.565	—	—	—	—

RC, mmol/L	1.251 (1.093–1.430)	0.001	1.461 (1.151–1.855)	0.002	—	—

RC						

Q1	**Reference**		—	—	**Reference**	

Q2	1.194 (1.026–1.391)	0.022	—	—	1.193 (1.015–1.402)	0.032

Q3	1.398 (1.204–1.623)	<0.001	—	—	1.356 (1.152–1.596)	<0.001

Q4	1.436 (1.238–1.666)	<0.001	—	—	1.404 (1.164–1.694)	<0.001


BMI, body mass index; ACS, acute coronary syndrome; COPD, chronic obstructive pulmonary disease; CHD, coronary heart disease; PCI, percutaneous coronary intervention; CABG, coronary artery bypass graft; LVEF, left ventricle ejection fraction; PLT, platelet count; MPV, mean platelet volume; LDL-C, low-density lipoprotein cholesterol; HDL-C, high-density lipoprotein cholesterol; TC, total cholesterol; RC, remnant cholesterol; eGFR, estimated glomerular filtration rate; CCB, calcium channel blocker; MA(ADP), adenosine diphosphate (ADP)-induced platelet maximum amplitude; LTPR, low on-treatment platelet reactivity; HTPR, high on-treatment platelet reactivity.

In the further analysis, based on the quartiles of RC, compared to Q1, after adjusting for the same significant covariates in HTPR model, the multivariable logistic regression analysis showed that Q4 had the highest risk of HTPR (OR: 1.404, 95% CI 1.164–1.694; p < 0.001), followed by Q3 (OR: 1.356, 95% CI 1.152–1.596; p < 0.001) and Q2 (OR: 1.193, 95% CI 1.015–1.402; p = 0.032) (Table 3).

### 3.3. Subgroup analysis

**Figure 3:**
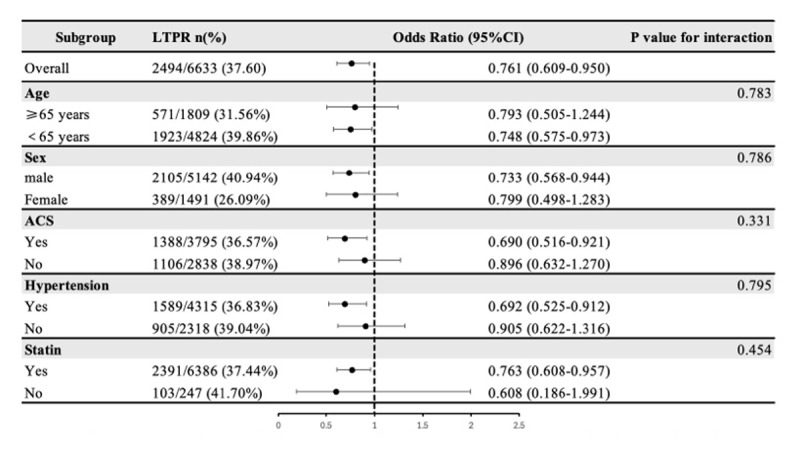
Subgroup analysis of the association between remnant cholesterol and LTPR.

**Figure 4:**
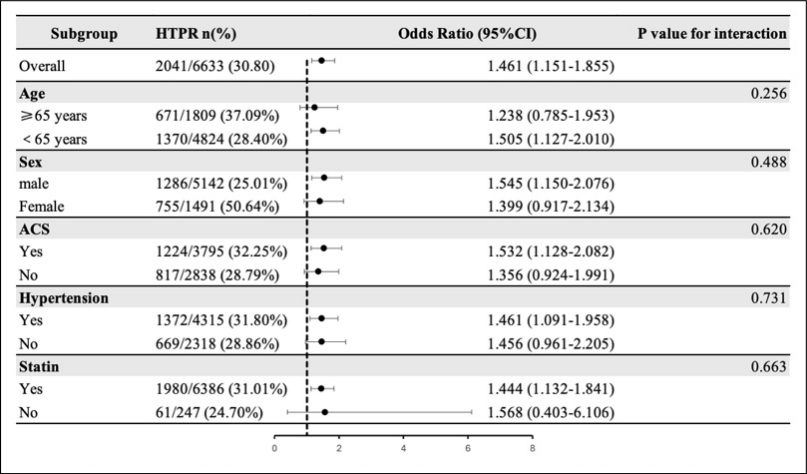
Subgroup analysis of the association between remnant cholesterol and HTPR.

The association of RC concentration with LTPR and HTPR both showed no significant interaction with whether age (≥65 years), sex, ACS, hypertension and the use of statin (P-value for interaction > 0.05) (Figures 3 and 4).

## 4. Discussion

This study, based on a large real-world cohort (n = 6,633), yielded several important findings. RC concentrations were associated with clopidogrel-related platelet reactivity in patients undergoing PCI received dual antiplatelet therapy; Specifically, RC concentration is negatively correlated with LTPR, meaning that the lower the RC concentration, the higher the risk of LTPR; meanwhile, RC concentration is positively correlated with HTPR, meaning that the higher the RC concentration, the higher the risk of HTPR. This may influence the bleeding and ischemic risks in patients treated with clopidogrel after PCI.

### 4.1. RC, LTPR, and bleeding

RC, as the cholesterol content of triglyceride-rich lipoproteins, was composed of very-low and intermediate density lipoproteins in the fasting state and chylomicron remnants in the nonfasting state (15). In recent years, the association between RC and the prognosis of individuals at high risk for cardiovascular events has attained significant attention. The relationship between RC and LTPR in patients under PCI remains unexplored in the existing literature.

Our study found that lower RC levels were associated with an increased risk of LTPR in patients. It is hypothesized that lower RC levels may increase the bleeding risk in patients by reducing platelet reactivity. This finding is of considerable importance in managing the bleeding risk of PCI patients with clopidogrel. However, the association between RC and bleeding has been seldom analyzed in the literature: Wu et al. found a relatively linear correlation between RC and bleeding risk, with lower RC levels linked to bleeding events during the acute phase of ischemic stroke and TIA (5). Our previous study also demonstrated that lower RC concentrations, rather than lower LDL-C concentrations, were independently associated with long-term bleeding risk in PCI populations (4). Thus, this study further confirms the correlation between RC and bleeding risk, which that has not been sufficiently emphasized in previous research.

### 4.2. RC, HTPR, and ischemia

In this research, we also observed that in PCI patients receiving clopidogrel, high RC levels is associated with an elevated risk of HTPR. This finding suggested that elevated RC concentrations may contribute to an increased ischemic risk by enhancing platelet reactivity. Previous studies have established a clear association between increased RC concentrations and heightened risk of ischemia, contributing to conditions such as ischemic heart disease, myocardial infarction, and stroke (6,16). A Mendelian randomization study revealed that for per 1.0 mmol/L increase of RC, the risk of ischemic heart disease increased by 2.8 times, independent of LDL-C or HDL-C levels (17). Similarly, Liao et al. (18) found a positive correlation between elevated RC levels and ischemic events in patients with ACS undergoing PCI and receiving statin therapy. As RC concentrations increased, the risk of ischemic events rose correspondingly (18). Our findings provide new mechanistic insights, explaining from a platelet perspective how higher levels of RC may partially contribute to increased ischemic risk.

### 4.3. Potential mechanisms

RC has been identified as a significant pathogenic risk factor in the development of atherosclerotic cardiovascular disease (19). Lipid metabolism may interact with endothelial dysfunction, inflammatory responses, thrombosis, and bleeding mechanisms (20). Mochizuki et al. demonstrated that chylomicron remnants and VLDL remnants markedly enhance the platelet aggregation in healthy individuals (21). These remnants infiltrate the arterial intima, where they accumulate and contribute to the development of atherosclerosis (20). Atherosclerosis fosters a pro-inflammatory and pro-thrombotic environment, significantly impacting platelet reactivity. Inflammation compromises endothelial function, reducing its ability to prevent platelet adhesion and activation, thus increasing platelet reactivity and promoting thrombus formation. Additionally, pro-inflammatory cytokines and neutrophil extracellular traps further exacerbate platelet activation within atherosclerotic plaques (22,23). Upon rupture of an atherosclerotic plaque, the exposed subendothelial matrix triggers a further escalation in platelet reactivity, leading to aggregation and thrombus formation, which can result in acute ischemic events such as ACS or stroke (24).

Additionally, studies have found that lowering cholesterol levels could reduce platelet reactivity (25). Platelet signaling is affected by cholesterol depletion, as the free cholesterol content of platelets influences their adhesion, activation, and aggregation capacity. Cholesterol depletion leads to a reduction in mean platelet volume and decreased platelet reactivity (26). Further research is warranted to elucidate the mechanistic link between RC and platelet reactivity, providing insights into the role of RC in cardiovascular risk management and therapeutic interventions.

### 4.4. Clinical implications and future directions

Our study demonstrate that lower RC concentrations are independent predictors of LTPR in PCI patients, while higher RC concentrations are independent predictors of HTPR. This further validates the intricate association between lipids and thrombosis. It is believed that within the context of current lipid-lowering strategies, maintaining RC levels within a reasonable range and regularly monitoring RC concentrations with necessary adjustments is crucial. Incorporating RC monitoring into clinical practice may enhance the efficacy and safety of lipid-lowering and antiplatelet therapies. As a lipid biomarker, RC should receive greater attention in lipid management to optimize cardiovascular risk assessment and intervention strategies.

### 4.5. Limitations

This study has several limitations. First, as a single-center observational study, its generalizability may be restricted. Second, variations in platelet function assessment methods could affect the platelet reactivity. Third, the study was limited to patients receiving clopidogrel; additional studies are needed to assess whether these findings can be generalized to patients treated with other P2Y12 inhibitors. Fourth, the inclusion of both urgent and scheduled PCI patients may introduce heterogeneity in antiplatelet treatment and clinical outcomes, and further research is needed to address this. Last, a limitation of this study is the absence of data on bleeding or ischemic events during hospitalization or follow-up, which could have further contextualized our findings.

## 5. Conclusion

In this large sample real-world study, we first reported that lower RC concentration was an independent risk factor for LTPR, whereas higher RC concentration was an independent risk factor for HTPR. Our findings contribute to the understanding of how varying concentrations of RC may be associated with increased risks of bleeding and ischemia. In the future, RC could serve as an additional marker to enhance risk identification in CAD patients treated with clopidogrel, providing valuable information in clinical situations.

## Data Accessibility Statement

Owing to ethical restrictions related to the consent given by subjects at the time of study commencement, our datasets are available from the corresponding author upon reasonable request after permission of the Institutional Review Board of the State Key Laboratory of Cardiovascular Disease, Fuwai Hospital, National Center for Cardiovascular Diseases.

## Additional File

The additional file for this article can be found as follows:

10.5334/gh.1475.s1 Table S1. Baseline characteristics of patients according to the platelet reactivity.
