# Supplementary material for: Association of Remnant Cholesterol with Platelet Reactivity in Coronary Artery Disease Patients Receiving PCI
Source: Glob Heart. 2025 Oct 15;20(1):95. doi: 10.5334/gh.1475 (PMC12533439; doi:10.5334/gh.1475)
Supplement: Table S1. — Baseline characteristics of patients according to the platelet reactivity. [file gh-20-1-1475-s1.pdf]

Table S1: Baseline characteristics of patients according to the platelet reactivity

Parameter	LTPR MAADP<31mm N=2494	NTPR 31mm ≤MAADP≤47mm N=2098	HTPR MAADP>47mm N=2041	*P*-value
Demographics				
Sex [male,n(%)]	2105 (84.40)	1751 (83.46)	1286 (63.01)	<0.001
Age, year	57.081±10.047	57.856±10.380	59.910±10.145	<0.001
BMI, kg/m^2	25.977±3.214	26.007±3.120	25.964±3.164	0.904
Clinical characteristics				
ACS, *n* (%)	1388 (55.65)	1183 (56.39)	1224 (59.97)	<0.001
Smoking history, *n* (%)	1527 (61.23)	1263 (60.20)	979 (47.97)	<0.001
Hyperlipidemia, *n* (%)	1708 (68.48)	1437 (68.49)	1383 (67.76)	0.841
Hypertension, *n* (%)	1589 (63.71)	1354 (64.54)	1372 (67.22)	0.040
COPD, *n* (%)	53 (2.13)	46 (2.19)	48 (2.35)	0.872
Family history of CHD, *n* (%)	622 (24.94)	523 (24.93)	495 (24.25)	0.838
Cerebrovascular disease history, *n* (%)	232 (9.30)	226 (10.77)	229 (11.22)	0.081
Peripheral vascular disease, *n* (%)	71 (2.85)	51 (2.43)	68 (3.33)	0.221
Prior myocardial infarction, *n* (%)	526 (21.09)	438 (20.88)	349 (17.10)	0.001
Prior PCI, *n* (%)	660 (26.46)	517 (24.64)	451 (22.10)	0.003
Prior CABG, *n* (%)	99 (3.97)	99 (4.72)	73 (3.58)	0.167
Laboratory variables				
LVEF, %	63.210±7.043	62.748±7.242	62.750±7.286	0.041
Hemoglobin, g/L	146.669±14.466	143.857±14.356	136.763±15.016	<0.001
PLT, 10^9/L	201.394±53.651	198.848±51.368	216.599±57.763	<0.001
MPV, fL	10.617±0.915	10.603±0.921	10.585±0.904	0.508
LDL-C, mmol/L	2.420±0.878	2.463±0.903	2.587±0.892	<0.001
HDL-C, mmol/L	1.028±0.270	1.020±0.267	1.035±0.274	0.2045
TC, mmol/L	4.114±1.042	4.154±1.070	4.324±1.069	<0.001
RC, mmol/L	0.667±0.360	0.672±0.391	0.702±0.377	0.004
RC				<0.001
Q1	677 (27.15)	560 (26.69)	440 (21.56)	
Q2	612 (24.54)	525 (25.02)	483 (23.66)	
Q3	600 (24.06)	508 (24.21)	551 (27.00)	
Q4	605 (24.26)	505 (24.07)	567 (27.78)	
Triglyceride, mmol/L	1.777±1.062	1.755±1.084	1.800±0.998	0.390
Glucose, mmol/L	5.989±1.821	6.054±1.903	6.260±2.123	<0.001
eGFR, ml/min	92.663±14.256	92.244±14.428	89.863±15.420	<0.001
Medication				
statin	2391 (95.87)	2015 (96.04)	1980 (97.01)	0.103
β-blockers	2248 (90.14)	1900 (90.56)	1862 (91.23)	0.453
CCB	1292 (51.80)	1029 (49.05)	1016 (49.78)	0.150
Platelet reactivity				
MA(ADP), mm	16.698±8.811	39.616±4.564	55.651±6.097	<0.001

BMI, body mass index; ACS, acute coronary syndrome; COPD, chronic obstructive pulmonary disease; CHD, coronary heart disease; PCI, percutaneous coronary intervention; CABG, coronary artery bypass graft; LVEF, left ventricle ejection fraction; PLT, platelet count; MPV, mean platelet volume; LDL-C, low-density lipoprotein cholesterol; HDL-C, high-density lipoprotein cholesterol; TC, total cholesterol; RC, remnant cholesterol; eGFR, estimated glomerular filtration rate; CCB, calcium channel blocker; MA(ADP), adenosine diphosphate (ADP)-induced platelet maximum amplitude; LTPR, low on-treatment platelet reactivity; HTPR, high on-treatmet platelet reactivity.
